# Modelling Mutation in Equine Infectious Anemia Virus Infection Suggests a Path to Viral Clearance with Repeated Vaccination

**DOI:** 10.3390/v13122450

**Published:** 2021-12-06

**Authors:** Elissa J. Schwartz, Christian Costris-Vas, Stacey R. Smith?

**Affiliations:** 1 Department of Mathematics & Statistics, School of Biological Sciences, Washington State University, Pullman, WA 99164, USA; ejs@wsu.edu; 2 Department of Mathematics, The University of Ottawa, Ottawa, ON K1N 6N5, Canada; ccost069@uottawa.ca; 3 Department of Mathematics and Faculty of Medicine, The University of Ottawa, Ottawa, ON K1N 6N5, Canada

**Keywords:** equine infectious anemia virus, mutation, vaccination, antibody infusion, mathematical model, impulsive differential equations

## Abstract

Equine infectious anemia virus (EIAV) is a lentivirus similar to HIV that infects horses. Clinical and experimental studies demonstrating immune control of EIAV infection hold promise for efforts to produce an HIV vaccine. Antibody infusions have been shown to block both wild-type and mutant virus infection, but the mutant sometimes escapes. Using these data, we develop a mathematical model that describes the interactions between antibodies and both wild-type and mutant virus populations, in the context of continual virus mutation. The aim of this work is to determine whether repeated vaccinations through antibody infusions can reduce both the wild-type and mutant strains of the virus below one viral particle, and if so, to examine the vaccination period and number of infusions that ensure eradication. The antibody infusions are modelled using impulsive differential equations, a technique that offers insight into repeated vaccination by approximating the time-to-peak by an instantaneous change. We use impulsive theory to determine the maximal vaccination intervals that would be required to reduce the wild-type and mutant virus levels below one particle per horse. We show that seven boosts of the antibody vaccine are sufficient to eradicate both the wild-type and the mutant strains. In the case of a mutant virus infection that is given infusions of antibodies targeting wild-type virus (i.e., simulation of a heterologous infection), seven infusions were likewise sufficient to eradicate infection, based upon the data set. However, if the period between infusions was sufficiently increased, both the wild-type and mutant virus would eventually persist in the form of a periodic orbit. These results suggest a route forward to design antibody-based vaccine strategies to control viruses subject to mutant escape.

## 1. Introduction

Equine infectious anemia virus (EIAV) is a lentivirus that primarily infects horses and shares many characteristics with HIV, including its viral structure, genome, life cycle and transmission via blood. EIAV establishes a persistent infection and is transmitted between hosts by biting flies [1]; the virus is prevalent in warmer climates, although not exclusively so [2].

EIAV infection is a substantial concern for equine health worldwide—one of only 11 infections of equids requiring reporting to the OIE, the World Organization for Animal Health [3]. EIAV infection has a global distribution, and recent reports have documented EIAV outbreaks in North America, South America, Asia and throughout Europe [3,4,5,6,7]. Genetically diverse strains have been found; thus far, phylogenetics has identified six clades globally, though the extent of EIAV diversity is expected to be higher in actuality [6].

The course of infection typically consists of three stages: an acute stage characterised by high fever and thrombocytopenia (i.e., low platelet count); a chronic stage with spiking viral loads, recurring febrile episodes and wasting; and an asymptomatic phase demonstrating a decreased viral load and an absence of apparent clinical symptoms [8]. There is no treatment for animals infected with EIAV.

In many areas, preventing transmission is managed by quarantine or euthanasia of animals that test positive for infection, with testing either regularly mandated or occurring when animals are moved or change ownership [4]. There are no prophylactic or therapeutic vaccines in current use against EIAV, though the control of EIAV in China has been attributed to a live-attenuated vaccine employed there between 1975 and 1990 [4,5].

Severe combined immunodeficiency (SCID) in horses is a condition that, due to a naturally occurring defect in lymphoctye development, eliminates the horse’s ability to create adaptive immune responses, such as antibodies or cytotoxic T lymphocytes (CTLs) [9,10]. The infusion of plasma from long-term EIAV-infected immunocompetent horses into SCID horses, however, can endow them with protection from EIAV infection [11,12]. Understanding EIAV infection in both immunocompetent and SCID horses has produced some interesting results on immunological control of lentivirus replication and disease and on the nature and role of virus mutation in persistence and pathogenesis [11,12]. These studies are of interest in the context of HIV and efforts to develop an HIV vaccine [13]. One of the reasons these viruses are difficult to control is because mutation allows them to escape from therapies and immune responses. We need to better understand how antibodies can control EIAV infection when the virus mutates. There is a gap in knowledge concerning whether additional antibody infusions could prevent mutant escape, and if so, how many vaccine doses are needed to eliminate the mutant virus.

In a study by Taylor et al., plasma containing EIAV-specific antibodies from an infected horse was adoptively transferred to SCID horses experimentally infected with EIAV [11,12]. When infused prior to and after EIAV challenge, the plasma afforded clinical protection against EIAV-induced disease in all horses and explicitly prevented infection in one of them. It was thus established that the consignment of EIAV-specific antibodies to a SCID horse can protect against homologous EIAV challenge [11].

Vaccination of horses by antibody infusion is not a treatment under development for EIAV; these studies by Taylor et al. were undertaken to gain insights into vaccine development for HIV infection. While infused SCID horses can be protected from EIAV challenge with homologous strains, EIAV variants have arisen that are not controlled [11]. Thus, the escape of lentiviruses from broadly neutralizing antibodies (NAbs), as well as control by NAbs of heterologous infection, are not fully understood. For dynamical interactions such as these, a modelling approach can be highly instructive.

Previous EIAV modelling studies investigated the mechanics of viral infection [14], considering uninfected and infected cells, viruses, antibodies and CTL responses [15] or the absence of adaptive immune responses [16]. Schwartz et al. described infection in the context of sensitive or NAb-resistant strains, both in the presence of EIAV-specific antibodies [17] and with different modes of transmission [18], in order to better quantify lentivirus escape from antibody responses [19]. Geethamalini and Balamuralitharan studied a variety of theoretical models for EIAV and focused on mathematical analyses to determine semianalytical solutions and global stability of equilibria using homotopy analysis, [20] to estimate parameters [21] and to show the existence of a Hopf bifurcation [22].

We analyse a model of EIAV with ongoing mutation from a wild-type (NAb-sensitive) strain to a mutant (NAb-resistant) strain and an explicit relationship between growth and antibody control. Our aim is to determine whether repeated vaccinations through more antibody infusions can reduce the wild-type and mutant strains of the virus below one particle, and if so, how to determine the ideal vaccination period to ensure eradication. In the studies by Taylor et al., the authors compared a physiological vaccine dose, a low vaccine dose and a high vaccine dose. The vaccination regime they used consisted of three infusions, on days −1, 7 and 14 days post-infection.

For simplicity, we refer to the predominant strain of the virus inoculum as the wild-type strain and the antibody-neutralisation-resistant variant as the mutant [19]. The mutant has a reduced growth rate but is less susceptible to antibody control. As in our earlier work [23], we supplement our continuous model with discrete antibody infusions.

## 2. Methods

Our model is a special case of our earlier work [23], using logistic growth for both the wild type strain (notated by state variable *W*) and the mutant strain (notated by state variable *M*) [24,25]. Our model describes two important characteristics of viral behaviour in EIAV infection: (i) the potential of the virus to attain steady state when antibodies are absent and (ii) the possibility of viral eradication (i.e., reducing the virus level below one particle per horse) when antibodies are present. The logistic term also represents virus production with a carrying capacity or subject to target-cell limitation [26]. Calculation of the wild-type virus net growth rate (representing both growth and clearance and notated by parameter *r*) and the fitness cost of the mutation (notated by parameter *c*) was as in previous work [19]; this was achieved by fitting the model to data from EIAV-infected SCID horses.

The viral load was determined by RT-PCR on plasma vRNA samples collected prior to infection through 4–8 weeks after infection, with frequent sampling (i.e., every few days) [11,19]. Infected horses received infusions of antibodies via control or immune plasma before experimental virus infection.

In the current model, each strain of the virus has its own carrying capacity. The wild-type-virus carrying capacity (notated by parameter $K_{1}$) was calculated by fitting the data from EIAV-infected control SCID horses (i.e., without infusions of EIAV-specific antibodies), A2245, A2247, H707 and H713 [11], to the model equation for wild-type virus with no antibody neutralisation.

The mutant-virus carrying capacity (notated by parameter $K_{2}$) was determined by fitting data from infected EIAV-specific antibody-infused SCID horses, A2239 and A2240 [11], to the model equation for mutant virus with no antibody neutralisation. Curve fitting was performed using least squares parameter estimation with Berkeley Madonna software, using values (other than $K_{1}$ and $K_{2}$) as indicated in Table 1.

Antibodies (notated by state variable *A*) decay naturally at rate *d* and bind to the wild-type virus at rate *p* and the mutant at rate (1−q)p, where *q* is small but nonzero. No antibody-production term is included, because SCID horses are unable to produce antibodies; in our model, antibodies can only increase due to plasma infusions, which occur according to the fixed amount $A_{\inf}$ at regular (or possibly irregular) times $t_{k}$.

**Figure 1:**
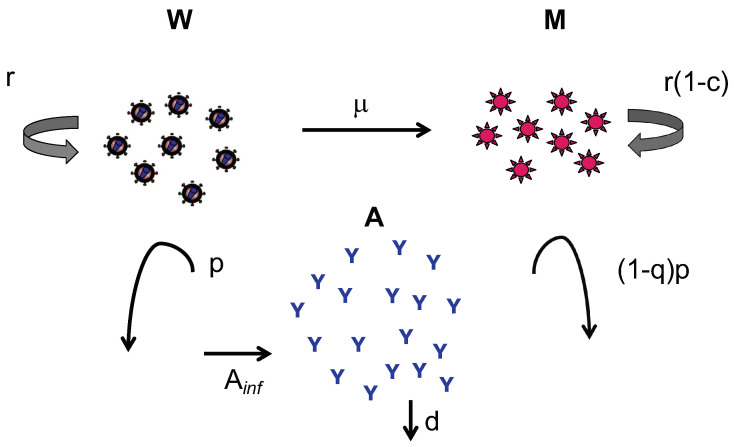
Schematic diagram of the model. The wild-type population (*W*) increases at rate *r*, mutates to the mutant strain at rate μ and is cleared by antibodies at rate *p*. The mutant population increases at rate r(1−c), where *c* is the fitness cost of the mutation; increases by mutation from the wild-type strain at rate μ; and is cleared by antibodies at rate (1−q)p, where (1−q) represents reduction in antibody blocking against the mutant. The antibody population increases at the time of each plasma infusion (by fixed amount $A_{\inf}$) and decays exponentially at rate *d*.

We model mutation via a continuous flow of the virus between the wild-type and mutant compartments at rate μ; the evolutionary cost for mutation is slower replication, represented by 1−c, as in [19]. This illustrates the evolutionary tradeoff for increased antibody resistance. The estimates of $A_{\inf}$, *p*, *d*, W(0), M(0), A(0) and μ (when multiplied by *r*; see model) are as in previous work [23]. The value of *q* is unknown, and thus a range of values (between 0 and 1) was taken in the simulations that follow; we took q=c as our sample value on the conjecture that the fitness loss of the mutant (*c*) is equal to its advantage gained in antibody insensitivity (*q*). A schematic diagram depicting our model is shown in Figure 1.

The model is then

(1) $$\begin{array}{cccc} W^{\prime } & =rW\left(1-\mu -\frac{W}{K_{1}}\right)-pAW\\ M^{\prime } & =r\left(1-c\right)M\left(1-\frac{M}{K_{2}}\right)+\mu rW-\left(1-q\right)pAM\\ A^{\prime } & =-dA & t & \neq t_{k}\\ \Delta A & =A_{\inf} & t & =t_{k}. \end{array}$$

Here, $W^{\prime }$, $M^{\prime }$ and $A^{\prime }$ indicate the time derivatives $\frac{dW}{dt}$, $\frac{dM}{dt}$ and $\frac{dA}{dt}$, respectively, while Δ stands for an instantaneous jump due to antibody infusion.

The model takes the form of impulsive differential equations [28,29,30]. For $t\neq t_{k}$, the model is continuous. At infusion times $t_{k}$, the antibody level is assumed to increase instantaneously. While this approximation assumes the time-to-peak is zero, such formulations are valid if the cycle time (the time between antibody infusions) is significantly larger than the actual time-to-peak [31]. The model parameters are given in Table 1.

## 3. Results

**Figure 2:**
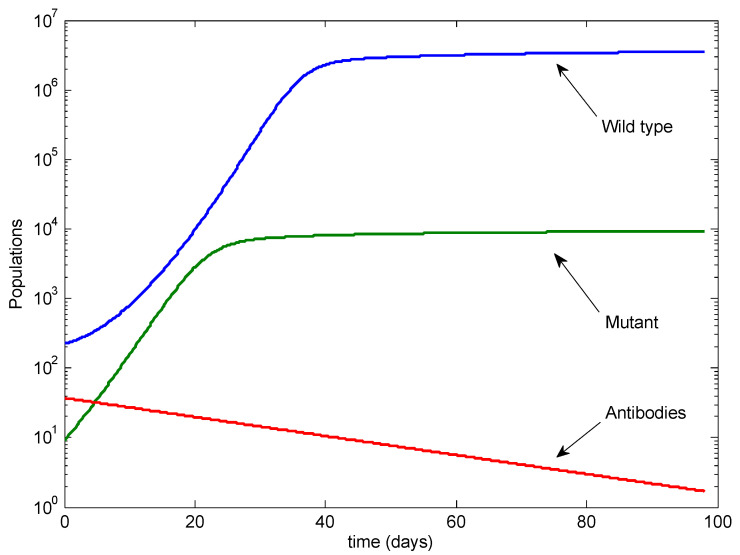
The baseline case with no antibody infusions after infection. Note the log scale on the vertical axis.

To illustrate the potential outcomes, we performed analytical calculations (see Appendix A) and simulated model (1) using the parameters from Table 1 unless otherwise specified. Figure 2 illustrates the baseline case, with only a single antibody infusion given at Day −1 and no doses following infection. In this case, the wild-type strain dominates, with the mutant at low levels.

**Figure 3:**
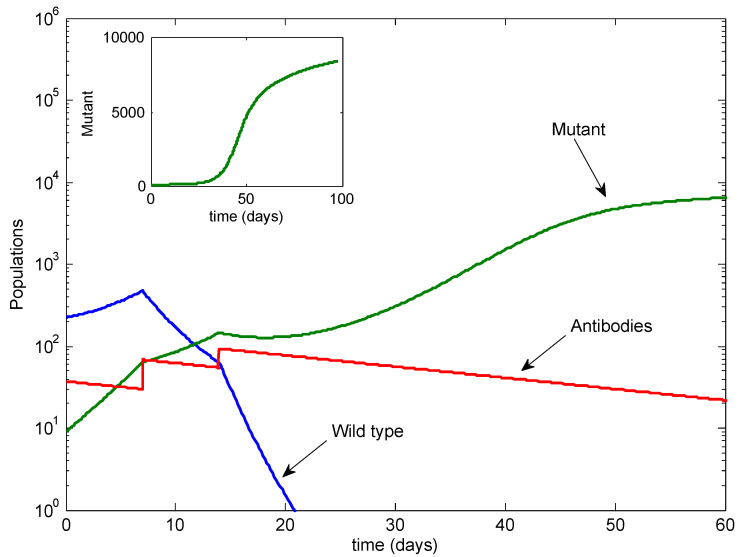
The standard case with three infusions. The wild type is cleared, but the mutant is not. Inset: the mutant on a linear scale.

Figure 3 illustrates the standard case of three antibody infusions, given at Day −1, Day 7 and Day 14, as per the protocol followed in Taylor et al. [11,12]. In this case, the wild-type strain is eradicated, while the mutant dominates. Note that the eventual mutant level is similar to the mutant level in Figure 2.

**Figure 4:**
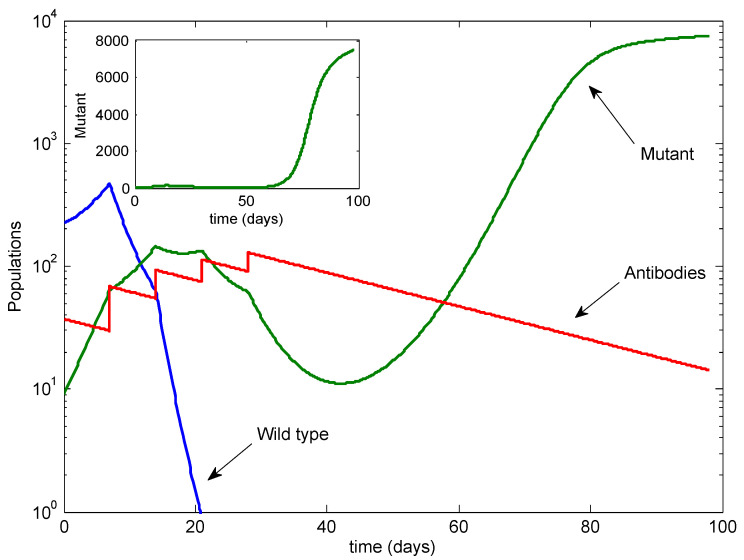
The case with two additional infusions. The mutant rebounds after the infusions stop.

Next, we examined the possibility of additional infusions; see Figure 4. With two additional infusions, at Days 21 and 28, the mutant was temporarily controlled but rebounded after the infusions stopped.

**Figure 5:**
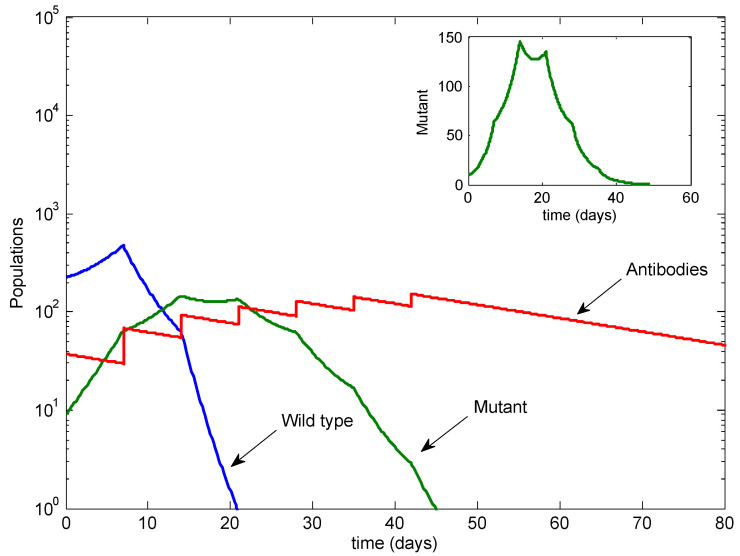
The case with four additional infusions. Both the wild type and mutant are cleared.

Additionally, we added infusions on Days 35 and 42, for a total of seven doses. See Figure 5. In this case, the mutant was also eradicated, eventually falling below 1 virus particle per horse, shortly after the last antibody infusion. In this case, the mutant peaked at only 150 viral particles.

**Figure 6:**
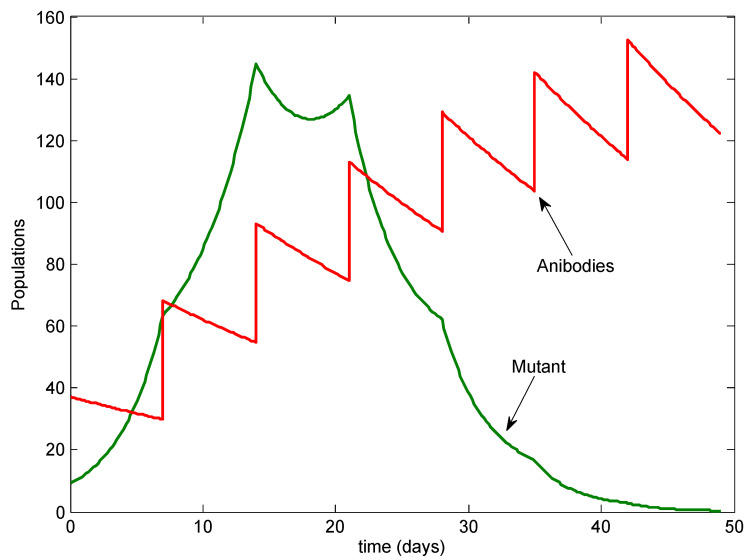
The case of mutant-only virus infection with the same antibodies against wild-type virus as before (i.e., heterologous infection). With four additional antibody infusions, the mutant is cleared.

We then investigated the case of control of heterologous infection by NAbs (i.e., infection with mutant virus but with infusions of antibodies against wild type as earlier) by setting W(0)=0 and M(0)>0. In this case, we determined that seven antibody infusions are likewise required for viral eradication. See Figure 6.

**Figure 7:**
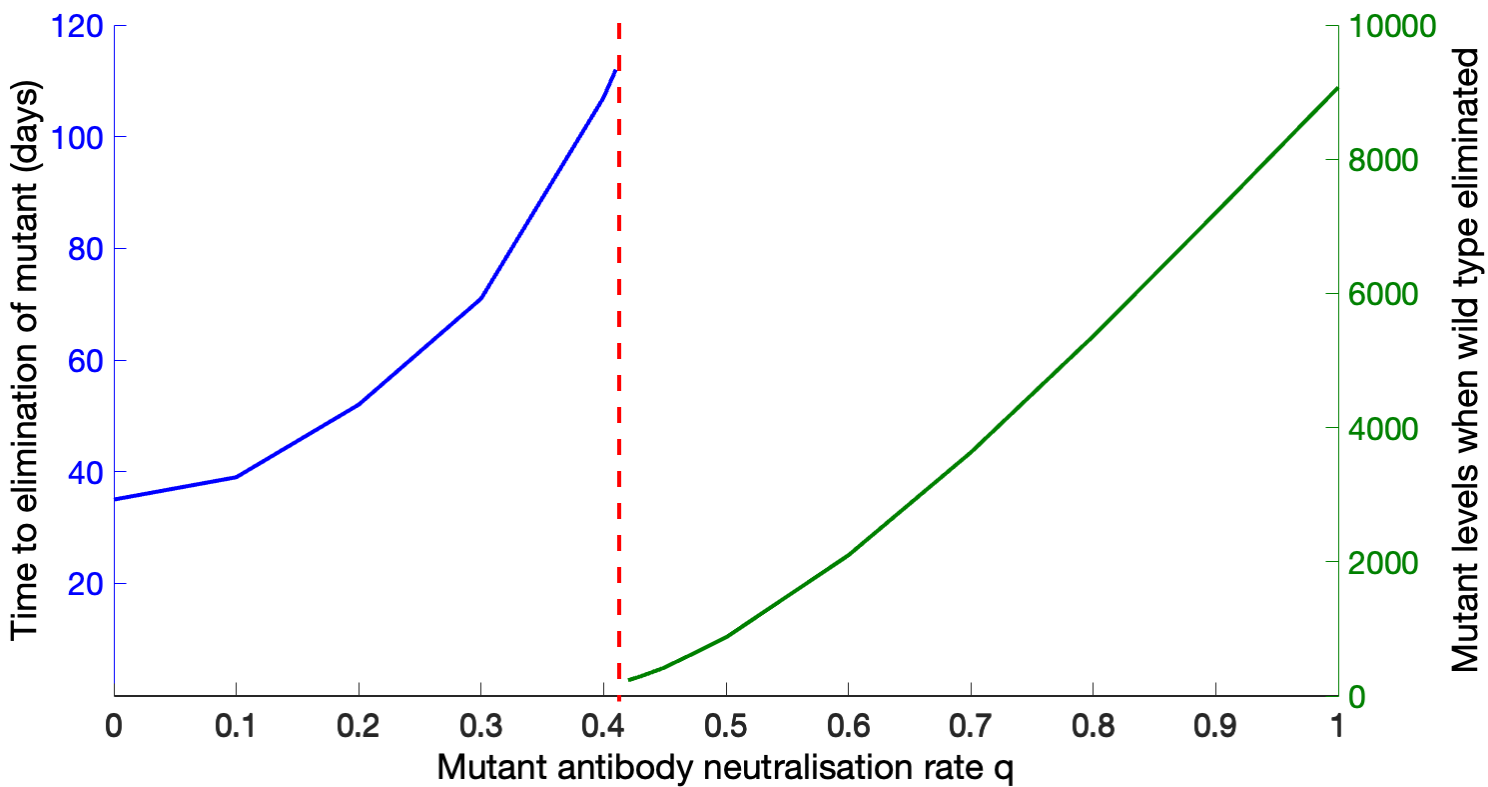
Sensitivity of the outcome to the mutant antibody-neutralisation rate, *q*. Below q=0.41, elimination of the mutant is possible; above q=0.41, elimination is no longer possible, and the mutant levels increase as *q* increases.

We also examined the sensitivity of our results to the parameter *q*, since data were not available. We varied *q* between 0 and 1 with all other values as in Table 1 and found a switch in outcomes: below q=0.41, the mutant is eliminated, although the time to elimination increases as *q* increases; beyond q=0.41, elimination is not possible, and the mutant values at the time of wild-type elimination increase as *q* increases until they reach the carrying capacity of the mutant. See Figure 7. If, for example, *q* and *c* are of similar values, then the mutant can be eliminated, which occurs after about 50 days. Alternatively, if *q* is four-fold higher than *c*, then the mutant cannot be eliminated, although the wild type is. At the time of wild-type elimination, the mutant levels reach approximately half the mutant carrying capacity.

The *q*-threshold is only mildly sensitive to other parameters. If the wild-type and mutant carrying capacities are identical, then the *q*-threshold moves slightly earlier, to 0.399 instead of 0.41. In the (extreme) case that c=0, the *q*-threshold moves earlier, to 0.3 instead of 0.41.

**Figure 8:**
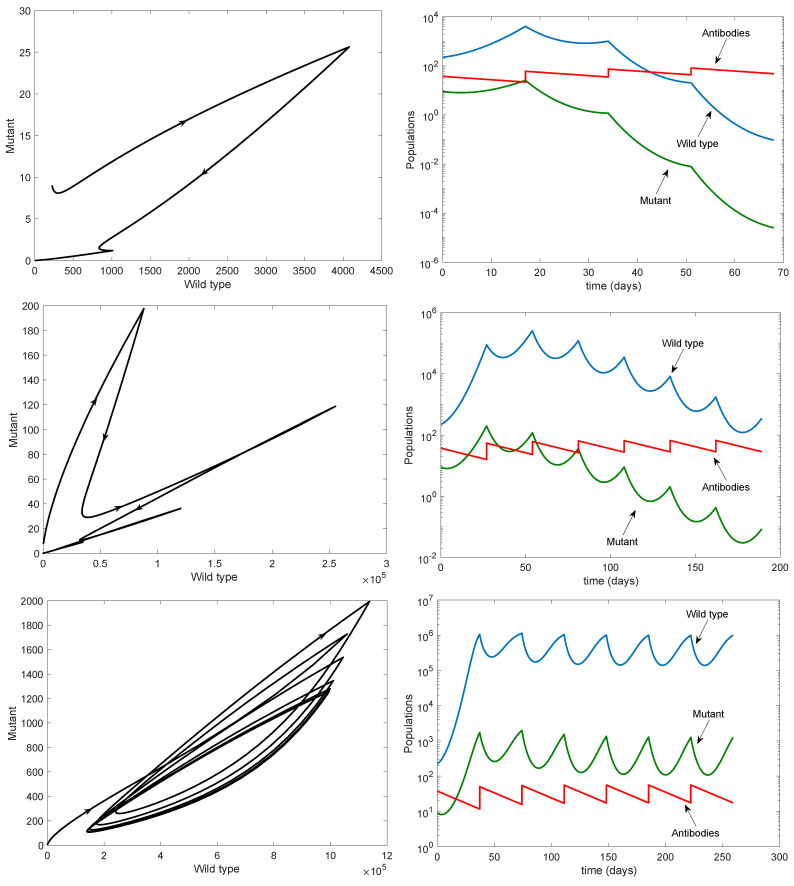
Effects of changing periods on the persistence of the wild type and mutant strains. All parameters are as in Table 1 except for the period. Left column: phase plane; right column: time series (note the log scale). Top row: τ=17. Middle row: τ=27. Bottom row: τ=37. Note the different scales on the axes.

Finally, we illustrated the effect of changing the period on the persistence of both strains. The top row of Figure 8 shows that both strains can be eradicated when the period is small, with eradication occurring quickly for both strains. The middle row shows that the time to eradication increases as the period increases, with potentially large transient values of the wild-type virus occurring before eradication is achieved. The bottom row shows both strains persisting in the form of a periodic orbit; in this case, the disease cannot be eradicated with finitely many antibody infusions.

## 4. Discussion

The potential for antibody infusions to control EIAV holds great promise for the potential elimination of other lentiviruses, such as HIV. However, the utility of this approach is limited by the potential for mutant escape. We have demonstrated a proof-of-concept that, given a virus that can mutate continuously to a NAb-resistant mutant (i.e., a potential escape mutant), both strains can be eradicated with only a finite number of infusions. Here, we have shown that, while the standard three infusions of antibodies may not be sufficient to eliminate the mutant, four additional infusions would clear EIAV from the horse.

As well as showing the potential benefit of increasing the number of antibody infusions, we also determined the maximal period between infusions in order to guarantee eradication of both the wild-type and mutant viruses. This allows us to design vaccination schedules for not just the number of infusions but also their frequency. The standard period of 7 days is sufficient to eradicate the virus with finitely many infusions; however, if the period is increased, then the time to eradication grows until eventually it cannot be achieved (see Figure 8).

These results were based on data from SCID horses with carrying capacities fitted to the antibody-free wild-type and mutant virus equations. The carrying capacity estimates differed by 3 logs. This can be understood biologically in that the wild-type strain has the greatest prevalence in the population, and it has a selective advantage over the mutant strain. The mutant has reduced competitive ability in the presence of the wild type, and it only dominates when the wild type is inhibited (in this case, by the antibodies). Indeed, in Taylor et al., the ratio of the wild-type virus to the escape variant in the inoculum was found to be 24:1 [11].

Other parameter values of the model were obtained by fitting data from clinical EIAV infections [11,12,16,19,23]. The parameter *q*, measuring the antibody-neutralisation rate of the mutant, was unknown, and thus we set q=c, where *c* is the fitness cost of the mutant, on the premise that the fitness lost equals the NAbs-escape advantage. However, we performed a sensitivity analysis using the range of *q* values from 0 to 1 and found little variation in the outcome.

Our model has several limitations, which should be acknowledged. The interaction between antibodies and virus takes the form of mass action, which assumes a well-mixed process. We model the decay of antibodies exponentially, which is a good approximation for larger antibody populations but can be less accurate as the availability dwindles. We restricted our modelling to a single mutant, without evolution of new mutants or back mutation. Finally, the impulsive assumption approximates the antibody time-to-peak by zero; such assumptions are valid if the time approximated is short compared to the time between impulses, which is true in our case.

This study is a contribution to the body of existing work on the dynamics of wild-type and mutant viruses, much of which has focused on antiviral therapy and the emergence of drug-resistant variants. Canini et al. explored the dosage and duration of antiviral therapy for influenza A virus, that was needed to lower the risk of drug resistance, and found that the emergence of resistant variants was greater with lower doses of the drug [32].

Handel et al. used a stochastic model with ongoing evolution to assess the emergence of drug resistance when antiviral therapy was utilized to control an influenza pandemic [33]. They found that containment of a wild-type outbreak, as well as prevention of emergence of resistance, were possible with rapid and strong treatment. Smith and Wahl developed the first models of impulsive differential equations for infectious diseases, showing that sufficiently frequent dosing could theoretically maintain HIV viral loads below the level of detection even if resistance was present [34,35].

Finally, Rong et al. investigated the emergence of drug resistance in HIV-1 infection with antiretroviral therapy and specifically examined the resistance level of the mutant variant; their modelling results showed that the mutant will emerge and dominate more quickly when the resistance level is higher, which will also give rise to higher mutant viral loads [36].

Our results presented here are analogous to this previous work on antiviral therapy and the emergence of drug-resistant variants. Focusing on EIAV infection, we showed that a greater number of antibody infusions would be sufficient to not only eliminate wild type virus but also block the emergence of NAb-resistant mutant virus. Furthermore, the higher the mutant’s level of resistance to neutralisation by the infused antibodies was, the longer the time needed to eliminate the mutant and the greater the mutant viral load when elimination is no longer possible.

In this work, we calculated the maximum period between infusions in order to ensure the eradication of both the wild-type and NAb-resistant mutant virus and also showed that both can be eradicated using the standard vaccination regimen but with four additional antibody infusions. This suggests a route forward for viral control not only of EIAV but for other virus infections in which escape by neutralisation-resistant mutants is a concern.

## Figures and Tables

**Table 1:** Parameter values and initial conditions.

Parameter	Description	Sample Value	Units	Reference
*r*	Net virus growth rate	0.58	day ${}^{-1}$	[19,23]
μ	Mutation rate	$4.655\times 10^{-5}$	–	[23,27]
$K_{1}$	WT carrying capacity	$2.000\times 10^{6}$	virus/mL	Fitted
*p*	Antibody-neutralisation rate of WT	0.0142	mL/(mg·day)	[23]
*c*	Fitness cost of mutant	0.19	–	[19]
$K_{2}$	Mutant carrying capacity	$4.732\times 10^{3}$	virus/mL	Fitted
*q*	Ab neutralisation rate of mutant	0.19	–	Variable
*d*	Antibody decay rate	0.0315	day ${}^{-1}$	[23]
$A_{\inf}$	Antibody infusion	38.4	mg/mL	[11,12,23]
τ	Period	7	days	Variable
*k*	Number of infusions	3	–	Variable
$t_{k}$	time of the *k*th infusion	7	days	[11,12]
W(0)	Initial wild-type levels	224	virus/mL	[23]
M(0)	Initial mutant levels	9	virus/mL	[23]
A(0)	Initial antibody levels	37.2	mg/mL	[23]

## Appendix A. Theoretical Calculations

### Appendix A.1. Upper and Lower Bounds

Solving impulsive differential equations requires solving a set of nonlinear differential equations in finite time, which is not possible in general. Instead, we can determine the upper and lower bounds for each state variable and apply the impulsive effect.

#### Appendix A.1.1. Antibody Equations

Since we have

$$\begin{array}{cc} A^{\prime } & =-dA \end{array}$$

between each impulse, it follows that

$$\begin{array}{cc} A(t) & =A\left(t_{k}^{+}\right)e^{-d(t-t_{k})}. \end{array}$$

for $t_{k}\leq t\leq t_{k+1}$, where $t_{k}$ is the moment of the *k*th impulse.

Applying initial conditions recursively within each cycle, the population of antibodies satisfies

$$\begin{array}{cc} A(0) & =A_{0}\\ A(t_{1}^{-}) & =A_{0}e^{-dt_{1}}\\ A(t_{1}^{+}) & =A\left(t_{1}^{-}\right)+A_{\inf}\\ \; & =A_{0}e^{-dt_{1}}+A_{\inf}\\ A(t_{2}^{-}) & =\left(A_{0}e^{-dt_{1}}+A_{\inf}\right)e^{-d(t_{2}-t_{1})}\\ A(t_{2}^{+}) & =A\left(t_{2}^{-}\right)+A_{\inf}\\ \; & \vdots \end{array}$$

Thus, in the *k*th cycle, the population of antibodies can be described as

$$\begin{array}{cc} A(t) & =A\left(t_{k}^{+}\right)e^{-d(t-t_{k})}\\ \; & =\left[A\left(t_{k-1}^{+}\right)e^{-d(t_{k}-t_{k-1})}+A_{\inf}\right]e^{-d(t-t_{k})}. \end{array}$$

#### Appendix A.1.2. Wild-Type Virus Equations

In order to estimate the maximal periods, we define $A_{\min}$ to be the minimum antibody level while W(t)≥1. We use the upper bound of the change in *W* to find the corresponding limit of W(t):

$$\begin{array}{cc} W^{\prime } & \leq rW(1-\mu -W/K_{1}-pA_{\min}) \end{array}$$

Define $W_{k}^{+}\equiv W\left(t_{k}^{+}\right)$ to be the value of the wild-type virus immediately after the *k*th infusion and set the period to be $\tau =t_{k+1}-t_{k}$. Note that, since the impulse is only applied to the antibodies, $W_{k}^{-}=W_{k}^{+}$ for all *k*, and thus the wild-type virus is continuous at the impulse points, although its derivative is not.

We restrict our calculations to the case $K_{1}\left(1-\mu -pA_{\min}\right)-W_{0}>0$ (which is true for our parameters, even for a wide range $0\leq A_{\min}\leq A_{\inf}$) and assume that $K_{1}\gg 1$ (i.e., the carrying capacity of the wild-type virus is signficantly larger than one virus particle).

Separating variables, using partial fractions and integrating the above differential inequality, we have, for $t_{k}\leq t\leq t_{k+1}$,

$$\begin{array}{c} W\left(t\right)\leq \frac{K_{1}\left(1-\mu -pA_{\min}\right)W_{k}^{+}e^{r\left(1-\mu -pA_{\min}\right)\left(t-t_{k}\right)}}{K_{1}\left(1-\mu -pA_{\min}\right)-W_{k}^{+}+W_{k}^{+}e^{r\left(1-\mu -pA_{\min}\right)\left(t-t_{k}\right)}} \end{array}$$

since $K_{1}\left(1-\mu -pA_{\min}\right)-W_{k}^{+}>0$ in practice, since the wild-type virus levels before and hence after an infusion will still be less than the carrying capacity.

The impulsive effect here is trivial, since no impulse occurs in the overestimate. However, we can examine the wild-type overestimate as a semicontinuous inequality.

(A1) $$\begin{array}{cc} W_{1}^{+} & \leq \frac{K_{1}\left(1-\mu -pA_{\min}\right)W_{0}e^{r(1-\mu -pA_{\min})\tau }}{K_{1}\left(1-\mu -pA_{\min}\right)-W_{0}+W_{0}e^{r(1-\mu -pA_{\min})\tau }} \end{array}$$

(A2) $$\begin{array}{cc} W_{2}^{+} & \leq \frac{K_{1}\left(1-\mu -pA_{\min}\right)W_{1}^{+}e^{r(1-\mu -pA_{\min})\tau }}{K_{1}\left(1-\mu -pA_{\min}\right)-W_{1}^{+}+W_{1}^{+}e^{r(1-\mu -pA_{\min})\tau }} \end{array}$$

(A3) $$\begin{array}{cc} \; & =\frac{K_{1}\left(1-\mu -pA_{\min}\right)W_{0}e^{2r(1-\mu -pA_{\min})\tau }}{K_{1}\left(1-\mu -pA_{\min}\right)-W_{0}+W_{0}e^{2r(1-\mu -pA_{\min})\tau }} \end{array}$$

(A4) $$\begin{array}{cc} \; & \;\;\vdots \end{array}$$

(A5) $$\begin{array}{cc} W_{n}^{+} & \leq \frac{K_{1}\left(1-\mu -pA_{\min}\right)W_{0}e^{nr(1-\mu -pA_{\min})\tau }}{K_{1}\left(1-\mu -pA_{\min}\right)-W_{0}+W_{0}e^{nr(1-\mu -pA_{\min})\tau }}. \end{array}$$

Taking the limit, we have

(A6) $$\begin{array}{cc} \lim_{n\to \infty }W_{n}^{+} & \leq K_{1}\left(1-\mu -pA_{\min}\right). \end{array}$$

Note that this value matches the nontrivial equilibrium value from model (1) in the absence of antibodies. Hence, the overestimate is bounded by the mutation-adjusted carrying capacity.

We can calculate the ideal period, τ, that results in eradication under the condition that wild-type virus levels are kept below 1; i.e., $W_{n}^{+}\leq 1$; thus (noting that $1-\mu -pA_{\min}>0$ for our parameters), it follows from (A5) that

$$\begin{array}{cc} \; & K_{1}\left(1-\mu -pA_{\min}\right)W_{0}e^{nr(1-\mu -pA_{\min})\tau }\leq K_{1}\left(1-\mu -pA_{\min}\right)-W_{0} \end{array}$$

(A7) $$\begin{array}{c} \;+W_{0}e^{nr(1-\mu -pA_{\min})} \end{array}$$

(A8) $$\begin{array}{cc} \; & \tau \leq \frac{1}{nr(1-\mu -pA_{\min})}\ln\frac{K_{1}\left(1-\mu -pA_{\min}\right)-W_{0}}{K_{1}\left(1-\mu -pA_{\min}\right)W_{0}-W_{0}}. \end{array}$$

Note that the right-hand side of (A8) is only positive if $W_{0}<1$. However, since we are always infusing wild-type virus (i.e., $W_{0}\gg 1$), in practice, the right-hand side of (A8) will always be negative. It follows that this overestimate cannot guarantee eradication of the wild-type virus.

#### Appendix A.1.3. Mutant Virus Equations

Using the upper bound for the wild-type virus (A6), we can estimate the mutant upper bound as follows:

(A9) $$\begin{array}{cc} M^{\prime } & \leq r\left(1-c\right)M\left(1-\frac{M}{K_{2}}\right)+K_{1}\left(1-\mu -pA_{\min}\right)\mu r. \end{array}$$

Since this is quadratic in *M*, we can resolve the differential inequality using a standard integral. For $\beta^{2}-4\alpha \gamma >0$, we have

$$\begin{array}{cc} \int \frac{1}{\alpha x^{2}+\beta x+\gamma }dx & =\frac{1}{\sqrt{\beta^{2}-4\alpha \gamma }}\ln\left|\frac{2\alpha x+\beta -\sqrt{\beta^{2}-4\alpha \gamma }}{2\alpha x+\beta +\sqrt{\beta^{2}+4\alpha \gamma }}\right|+\bar{c}. \end{array}$$

Let

$$\begin{array}{cc} \delta & =2r\left(1-c\right)/K_{2}\\ \theta & =r(1-c)\\ \sigma & =\sqrt{r^{2}{\left(1-c\right)}^{2}+4r^{2}\left(1-c\right)\left(1-\mu -pA_{\min}\right)\mu K_{1}/K_{2}}\\ \bar{\sigma } & =\sqrt{r^{2}{\left(1-c\right)}^{2}-4r^{2}\left(1-c\right)\left(1-\mu -pA_{\min}\right)\mu K_{1}/K_{2}}. \end{array}$$

Using (A9), we can write

(A10) $$\begin{array}{cc} \ln\left|\frac{-\delta M+\theta -\sigma }{-\delta M+\theta +\bar{\sigma }}\right| & \leq \sigma \left(t-t_{k}\right)+\ln\left|\frac{-\delta M_{k}^{+}+\theta -\sigma }{-\delta M_{k}^{+}+\theta +\bar{\sigma }}\right| \end{array}$$

for $t_{k}\leq t\leq t_{k+1}$.

Note that we have the following relationships:

$$\begin{array}{cc} \sigma -\theta & >0\\ \sigma +\theta -\delta & >0\\ -\delta M_{0}+\theta -\sigma & >0. \end{array}$$

Since $-\delta M_{0}+\theta -\sigma <0$ and $M_{0}$ is small, it follows that −δM+θ−σ<0. Furthermore, we have $-\delta M_{0}+\theta +\bar{\sigma }>0$ since $M_{0}$ is small and $K_{2}\gg 1$. Then, using (A10), we have

$$\begin{array}{c} \delta M\left(1+e^{\sigma (t-t_{0})}\frac{\delta M_{0}-\theta +\sigma }{-\delta M_{0}+\theta +\bar{\sigma }}\right)\leq e^{\sigma (t-t_{0})}\left(\theta +\bar{\sigma }\right)\frac{eM_{0}-\theta +\sigma }{-\delta M_{0}+\theta +\bar{\sigma }}+\theta -\sigma \\ M\leq \frac{1}{\delta }\frac{\left(\theta -\sigma \right)\left(-\delta M_{0}+\theta +\bar{\sigma }\right)+\left(\theta +\bar{\sigma }\right)\left(\delta M_{0}-\theta +\sigma \right)e^{\sigma (t-t_{0})}}{-\delta M_{0}+\theta +\bar{\sigma }+e^{\sigma (t-t_{0})}\left(\delta M_{0}-\theta +\sigma \right)}. \end{array}$$

In particular,

$$\begin{array}{c} M_{1}^{+}\leq \frac{1}{\delta }\frac{\left(\theta -\sigma \right)\left(-\delta M_{0}+\theta +\bar{\sigma }\right)+\left(\theta +\bar{\sigma }\right)\left(\delta M_{0}-\theta +\sigma \right)e^{\sigma \tau }}{-\delta M_{0}+\theta +\bar{\sigma }+e^{\sigma \tau }\left(\delta M_{0}-\theta +\sigma \right)}. \end{array}$$

Since functions of the form

$$f\left(x\right)=\frac{-a+be^{x}}{c+de^{x}}$$

(with a,b,c,d>0) are increasing in *x* with upper limit $\frac{b}{d}$, it follows that

$$\begin{array}{cc} M_{1}^{+} & \leq \frac{1}{\delta }\left(\theta +\bar{\sigma }\right). \end{array}$$

Hence, by induction, we have $-\delta M_{n}^{+}+\theta +\bar{\sigma }\geq 0$ and

$$\lim_{n,\tau \to \infty }M_{n}^{+}\leq \frac{\theta +\bar{\sigma }}{\delta }.$$

It follows that $-\delta M+\theta +\bar{\sigma }>0$. Note also that, since M≥1 (or else there would be no mutant), we have

$$0<-\delta M+\theta +\bar{\sigma }\leq -\delta +\theta +\bar{\sigma }.$$

Applying the impulsive effect *n* times, we have

$$\begin{array}{c} M_{n}^{+}\leq \frac{1}{\delta }\frac{\left(\theta -\sigma \right)\left(-\delta M_{0}+\theta +\bar{\sigma }\right)+\left(\theta +\bar{\sigma }\right)\left(\delta M_{0}-\theta +\sigma \right)e^{n\sigma \tau }}{-\delta M_{0}+\theta +\bar{\sigma }+e^{n\sigma \tau }\left(\delta M_{0}-\theta +\sigma \right)}. \end{array}$$

The mutant will be eradicated if it falls below one virus particle. Hence, $M_{n}^{+}\leq 1$ implies

$$\begin{array}{cc} e^{n\sigma \tau }\left[\left(\theta +\bar{\sigma }-\delta \right)\left(\delta M_{0}-\theta +\sigma \right)\right] & \leq \left(-\delta M_{0}+\theta +\bar{\sigma }\right)\left(\delta +\sigma -\theta \right). \end{array}$$

Note that all terms in parentheses are positive. It follows that

(A11) $$\begin{array}{cc} \tau & \leq \frac{1}{n\sigma }\ln\frac{\left(-\delta M_{0}+\theta +\bar{\sigma }\right)\left(\delta +\sigma -\theta \right)}{\left(\delta M_{0}-\theta +\sigma \right)\left(\theta +\bar{\sigma }-\delta \right)}. \end{array}$$

This determines the maximal period between infusions in order to ensure the mutant is eradicated: if the period τ is chosen to satisfy (A11), then the mutant can theoretically be controlled using a finite number of antibody infusions.
